# Circulating MIF Associated With Disease Severity and Clinical Response of Sublingual Immunotherapy in House Dust Mite–Induced Allergic Rhinitis

**DOI:** 10.3389/fphar.2021.681724

**Published:** 2021-07-08

**Authors:** Shaobing Xie, Hua Zhang, Fengjun Wang, Zhihai Xie, Weihong Jiang, Kelei Gao

**Affiliations:** Department of Otolaryngology Head and Neck Surgery, Xiangya Hospital of Central South University and Hunan Province Key Laboratory of Otolaryngology Critical Diseases, Changsha, China

**Keywords:** macrophage migration inhibitory factor, sublingual immunotherapy, allergic rhinitis, house dust mite, clinical response, biomarker

## Abstract

**Background:** Macrophage migration inhibitory factor (MIF) is described as a pro-inflammatory cytokine involved in many inflammatory and allergic disorders, but the role of MIF in allergic rhinitis (AR) remains poorly clarified. The aim of this study was to investigate the association between circulating MIF levels and house dust mite (HDM)-induced AR, and evaluate MIF as a potential biomarker in reflecting disease severity and predicting the clinical response of sublingual immunotherapy (SLIT) in HDM-induced AR patients.

**Methods:** In this study, we enrolled 160 persistent HDM-induced AR patients (AR group), including 48 mild AR patients (MAR group) and 112 moderate–severe AR patients (MSAR group), and 77 healthy controls (HC group). Circulating levels of MIF were measured by ELISA, and the relationship between MIF concentrations and disease severity was assessed. In the MSAR group, 106 patients were assigned to receive SLIT for 3 years. At the end of the study, patients were categorized into good response group and poor response group, and associations between clinical variables or biomarkers and clinical response were analyzed by the multivariate regression analysis.

**Results:** The concentrations of serum MIF were significantly higher in AR patients than in HCs, especially in those with MSAR. Moreover, circulating MIF levels were positively correlated with TNSS, VAS, serum HDM–specific IgE, total IgE, blood eosinophil count, and blood eosinophil percentage (all *p* < 0.05). Eighty MSAR patients finally completed SLIT, 45 patients obtained good response, and 35 patients resulted in poor response. The serum levels of MIF were significantly lower in the good-response group than in the poor-response group (*p* < 0.001). The receiver operating characteristic analysis for MIF showed good accuracy for predicting clinical response of SLIT (area under the curve = 0.877, *p* < 0.001). The multivariate regression analysis demonstrated that serum MIF was an independent factor for SLIT responsiveness.

**Conclusion:** Serum MIF appeared to be an important biological indicator in reflecting disease severity and an independent predictor for clinical responsiveness of SLIT in HDM-induced AR patients.

## Introduction

Allergic rhinitis (AR) is a noninfectious inflammatory disease characterized by nasal manifestations, including nasal itching, nasal obstruction, rhinorrhea, and sneezing, caused by inhaled allergens such as dust mite, pollen, and animal hair (Zhang and Zhang, 2019; Bousquet et al., 2020). Previous studies reported that house dust mite (HDM) was prevalent worldwide and the most common cause of perennial AR, especially in the southern regions of China (Meng et al., 2019; Zhang and Zhang, 2019). Epidemiological studies showed that the prevalence of AR has increased progressively, and this clinical disorder approximately affected a billion people worldwide (Han et al., 2020; Meng et al., 2020). AR is highly heterogeneous with a wide range of severity, which has been subjectively evaluated by many symptom scoring scales, such as the total nasal symptom score (TNSS) and visual analogue scale (VAS) (Adamko et al., 2018; Shi et al., 2020). However, AR patients often do not realize how severe their symptoms are, and self-reported symptoms may hinder clinicians to evaluate the disease progression and adjust the treatment plan accordingly (Del Cuvillo et al., 2017; Han et al., 2019). Therefore, it is desired to explore promising and objective biomarkers with high sensitivity and specificity to facilitate the assessment of AR severity in clinical practice.

Presently, available treatment options for HDM-induced AR comprise patient education, allergen avoidance, pharmacotherapy, and allergen-specific immunotherapy (AIT) (Li et al., 2019; Zissler and Schmidt-Weber, 2020), but HDM AIT is the only effective cure in HDM-induced AR patients through modifying the natural course of disease and inducing immunological tolerance to the causal allergen (Meteran and Backer, 2019; Hoshino et al., 2020). AIT can be administrated subcutaneously (SCIT) or sublingually (SLIT), but an increasing number of patients tend to receive SLIT, due to its more convenience and less systemic reactions than those in SCIT (Li et al., 2018; Barker-Tejeda et al., 2020). Although SLIT has been demonstrated to be effective in AR, its efficacies differ between patients: some show no benefit even after prolonged treatment (Liu et al., 2020a; Zissler and Schmidt-Weber, 2020). Previous studies have explored several candidate indicators, such as serum-specific IgE (Kim et al., 2020), periostin (Hoshino et al., 2020), and serum metabolites (Xie et al., 2021), to predict the clinical efficacy of SLIT, but the sensitivity and specificity of these potential biomarkers are not satisfactory. Therefore, identifying specific biomarkers with high accuracy and reliability for predicting the clinical responsiveness of SLIT is urgently needed to support treatment decisions.

Macrophage inhibitory factor (MIF), a T lymphocyte–derived protein, is a pleiotropic inflammatory cytokine which is expressed in various cell types, including macrophages, T lymphocyte cells, eosinophils, and neutrophils (Zhao et al., 2019a; Bilsborrow et al., 2019; Gamez-Nava et al., 2020). Accumulating evidence showed that MIF exhibited a variety of biological functions in the inflammation and immune response, and was closely involved in allergic and autoimmune diseases (Yoshihisa et al., 2011; Bozza et al., 2020; Li et al., 2021). Previous studies demonstrated that the concentrations of MIF in serum and local tissue were significantly elevated in asthma (Lan et al., 2020; Li et al., 2021), atopic dermatitis (Yoshihisa et al., 2011), rheumatoid arthritis (Bilsborrow et al., 2019), and systemic lupus erythematosus (Gamez-Nava et al., 2020). Thus, it is reasonable to assume that MIF might also play an important role in the pathogenesis of AR. In the present study, we sought to investigate the changes of serum MIF levels in the HDM-induced AR patients and evaluate whether circulating MIF can serve as a promising biomarker in reflecting disease severity and predicting the clinical response of SLIT in HDM-induced AR patients.

## Methods

### Participants and Settings

In the present study, we consecutively enrolled 160 HDM-induced AR patients (AR group) who visited the Department of Otolaryngology Head and Neck Surgery, Xiangya Hospital of Central South University between the period of January 2017 and July 2017. Seventy-seven sex- and age-matched control subjects without any allergic diseases were recruited as the healthy control (HC) group. This study was conducted under the guidance of the Helsinki Declaration and approved by the Human Ethical Committee of Xiangya Hospital of Central South University, and all participants submitted written informed consent. HDM-induced AR was diagnosed by clinicians based on the allergic rhinitis and its impact on asthma (ARIA) guidelines (Brożek et al., 2017). The inclusion criteria were listed as follows: 1) persistent AR with typical allergic symptoms for more than 2 years, 2) a positive test result in nasal provocation, 3) a positive skin test to *Dermatophagoides farinae (Der f)* and/or *Dermatophagoides pteronyssinus (Der p)* (at least ++) and/or positive specific IgE against *Der f* and/or *Der p* (>0.35 IU/ml), and 4) age ≥18 years. The exclusion criteria were listed as follows: 1) active asthma that required inhaled corticosteroid treatment; 2) significant vasomotor rhinitis; 3) with other allergic diseases, inflammatory or septic diseases, or autoimmune diseases; 4) pregnant or planning pregnancy within the next three years; 5) severe cardiovascular diseases and impaired liver and renal function; 6) with an acute upper respiratory tract infection within 4 weeks; and 7) use of anti-allergic drugs within 4 weeks.

### Disease Symptoms Assessment

Patients were asked to record their disease symptoms with the visual analogue scale (VAS) and total nasal symptom score (TNSS) as previously described (Adamko et al., 2018; Zhao et al., 2019b). VAS is a 10-point scale from 0 (no symptom) to 10 (maximum). TNSS is the sum of four individual symptom scores (sneezing, rhinorrhea, nasal congestion, and nasal itching), and each symptom ranged from 0 to 3, resulting in a possible score of 0–12 (detailed described in Supplementary Table S1). Based on TNSS, AR patients were categorized into the mild AR (MAR) group (TNSS ≤4) and the moderate–severe AR (MSAR) group (TNSS >4) according to recommendation by previous publications (Refaat et al., 2015; Adamko et al., 2018).

### Sample Collection and Macrophage Inhibitory Factor Level Measurement

Five ml fresh venous blood were collected from AR patients and HCs before breakfast, and stored at room temperature for 1 h after sampling. Then, collected blood samples were centrifuged at 4°C (3,000 rpm for 10 min), and the supernatants were collected and stored at -80°C in 1.5 ml ep tubes (NEST Biotechnology, Wuxi, China) for subsequent experiments. All serum samples were coded before the measurements. Serum MIF concentrations were quantified with a commercial ELISA kit (Multisciences, Hangzhou, China) according to the manufacturer’s instructions. All serum samples were diluted 1:40 and run in duplicate to improve assay precision.

### Immunotherapy

In the MSAR group, 106 patients voluntarily received HDM SLIT for a period of 3 years to obtain long-term efficacy as previously suggested (Li et al., 2019). Eligible participants were administrated with the standardized *Der f* drops which were provided by Wolwopharma Biotechnology Company (Zhejiang, China) according to the schedules recommended by the manufacturer. SLIT consists of a step-up dosage phase and a maintenance phase, the detailed schedules were conducted as described in our previous study (Xie et al., 2021), and all adverse reactions were recorded.

### Follow-Up and Clinical Efficacy Evaluation

As patient compliance was an important factor affecting the efficacy of SLIT, all participants received patient education to improve their adherence as previously recommended, including initial education with publicity brochures (explaining the course, costs, efficacy, and safety of SLIT) at the first two months during the SLIT, and follow-up education through department visits or telephone (assessing patient’s recent clinical symptoms, adjusting the SLIT schedules, and guiding to avoid and manage adverse reactions) (Kiotseridis et al., 2018; Li et al., 2019). As SLIT cannot completely alleviate and even aggravate allergic symptoms during the treatment especially when allergen load is heavy, appropriate pharmacotherapy was provided to patients, including antihistamine and corticosteroid (Canonica et al., 2007; Li et al., 2019), and the medication scores (MSs) were recorded. The definition of MSs is the sum of medication consumption in the last week and scores according to the World Allergy Organization recommendations: oral or intranasal antihistamines = 1 point; nasal glucocorticoids = 2 point; and oral glucocorticoids = 3 point (Pfaar et al., 2014). The symptom and medication score (SMS) was calculated as the sum of TNSS and MS/7, and the efficacy of SLIT was evaluated on the basic of SMS as described in our previous publication; a change of >30% point compared to the baseline SMS level represents good response; otherwise, the SLIT was defined as poor response (Xie et al., 2020; Xie et al., 2021).

### Statistical Analysis

Normally distributed data were shown as mean ± standard deviation (SD), and non-normal distributed data were described as median and interquartile range. One-way analysis of variance (ANOVA) or the Mann–Whitney U test was utilized for comparison among three groups, and the subsequent comparisons were performed by Student–Newman–Keuls (SNK) to locate the source of significances; Student's t test or the Kruskal–Wallis H test was used for comparison between two groups. Categorical data were expressed as number (%) and compared utilizing the chi-square test. To investigate the correlations among MIF level, TNSS, VAS, and other clinical variables, Spearman’s test was conducted. The multivariate logistic regression analysis was performed to identify the independent predicting factors for the clinical responsiveness of SLIT. A receiver operating characteristic (ROC) curve was constructed to determine the utility of serum MIF and other indications as markers for predicting the efficacy of SLIT in given specimens, and the area under the curve (AUC), sensitivity, specificity, and cutoff value were evaluated. For all tests, *p* < 0.05 was considered statistically significant. All statistical analyses and ROC analysis were conducted by SPSS Statistics for Windows (Version 25.0, IBM Corp., Armonk, NY), and other figures were constructed in GraphPad Prism (Version 7.0, Software Inc. La Jolla, CA, United States).

## Results

### Basic Demographics and Characteristics of Participants

The basic demographic and clinical characteristics of all participants are displayed in Table 1. Age, sex, disease duration, BMI, and smoking were similar among three groups (*p* > 0.05). Patients in the MSAR group showed higher levels of serum total IgE, HDM-specific IgE, blood eosinophil count and percentage, TNSS, and VAS (all *p* < 0.05) than those in the HC and MAR groups.

**TABLE 1:** Demographic and clinical characteristics of participants among three groups.

Variables	HC (1) (n = 77)	MAR (2) (n = 48)	MSAR (3) (n = 112)	*p*-value	Comparison^a^
Age (year)	29.7 ± 8.2	29.0 ± 9.1	30.9 ± 8.4	0.157	1, 2, 3
Sex, male (%)	40 (51.9)	27 (56.3)	59 (52.7)	0.745	-
Disease duration (year)	NA	4.4 ± 1.8	4.5 ± 1.7	0.249	1, 2, 3
BMI (kg/m^2^)	22.8 ± 1.9	22.7 ± 1.7	22.9 ± 1.8	0.893	1, 2, 3
Smoking (%)	14 (18.2)	8 (16.7)	25 (22.3)	0.647	-
Serum total IgE (IU/ml)	78.3 (32.4, 112.5)	145.6 (84.9, 228.7)	247.4 (117.7, 486.2)	<0.001	1 < 2 < 3
Serum HDM–specific IgE (kU/L)	0.2 (0.1, 0.3)	12.8 (5.4, 21.6)	17.9 (8.5, 29.7)	<0.001	1 < 2 < 3
Blood eosinophil count (10^6^/L)	114.5 ± 38.9	223.6 ± 91.6	347.9 ± 124.6	<0.001	1 < 2 < 3
Blood eosinophil percentage (%)	1.5 ± 0.5	2.4 ± 1.1	3.8 ± 1.6	<0.001	1 < 2 < 3
TNSS	-	3 (2, 4)	9 (7, 11)	<0.001	-
VAS	-	3 (2, 4)	7 (5, 9)	<0.001	-

HC, healthy control; MAR, mild allergic rhinitis; MSAR, moderate–severe allergic rhinitis; BMI, body mass index; HDM, house dust mite; TNSS, total nasal symptom score; VAS, visual analogue scale.

a The subsequent comparisons were performed by Student–Newman–Keuls (SNK) to locate the source of significances.

### Migration Inhibitory Factor Levels in Allergic Rhinitis Patients and Healthy Controls and Correlations With Clinical Variables

**FIGURE 1:**
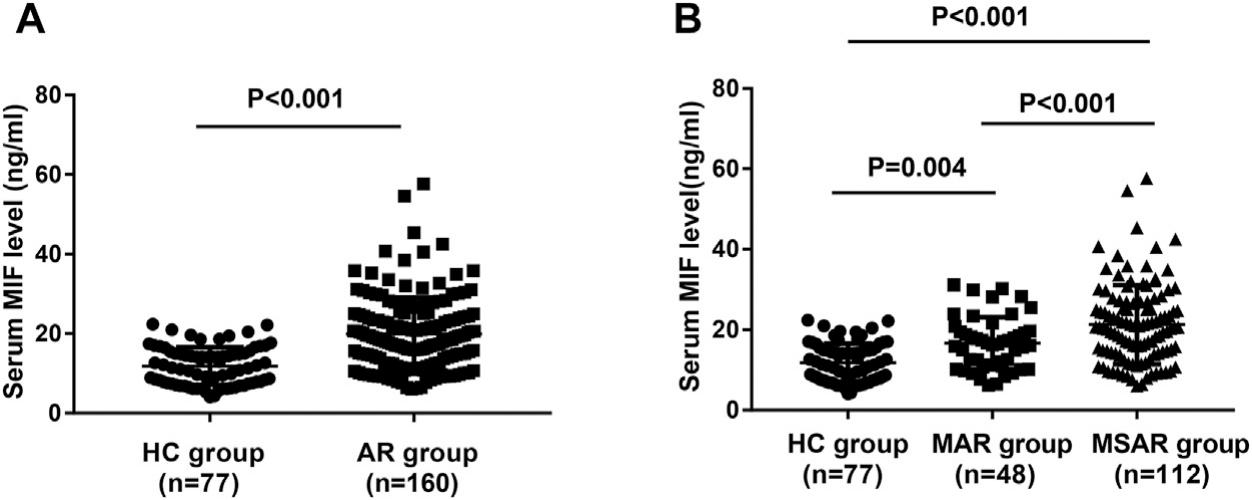
Comparison of serum MIF concentrations between HDM-induced AR patients and HCs. **(A)** MIF levels were significantly increased in the AR group than in the HC group. **(B)** Serum MIF levels were markedly elevated in the MSAR group than in the MAR group and HC group. MIF, macrophage migration inhibitory factor; HDM, house dust mite; AR, allergic rhinitis; HC, healthy control; MAR, mild allergic rhinitis; MSAR, moderate–severe allergic rhinitis.

**FIGURE 2:**
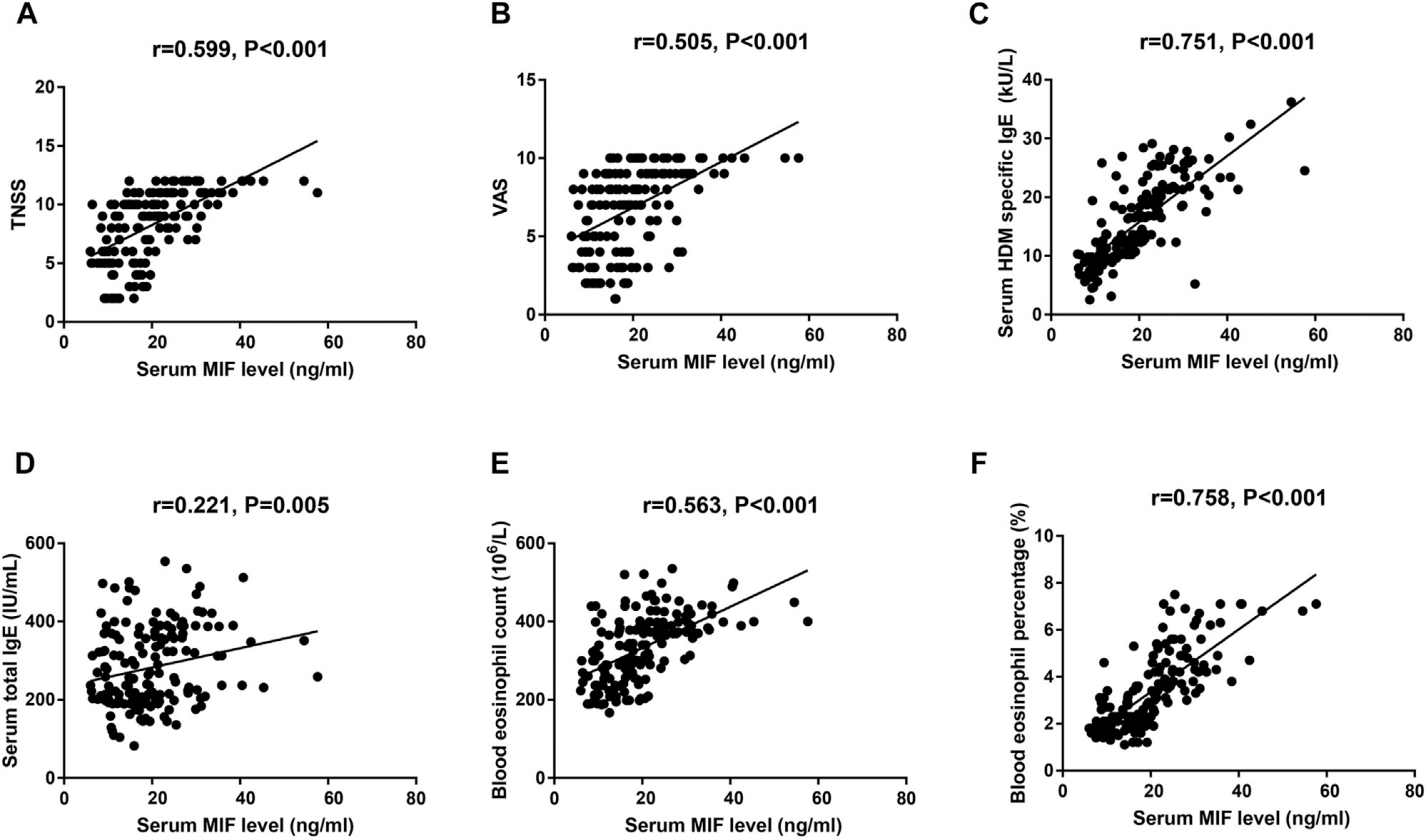
Spearman’s correlation analysis of association among serum MIF levels, TNSS **(A)**, VAS **(B)**, serum HDM–specific IgE **(C)**, total IgE **(D)**, blood eosinophil count **(E)**, and percentage **(F)** in HDM-induced AR patients. MIF, macrophage migration inhibitory factor; HDM, house dust mite; AR, allergic rhinitis; TNSS, total nasal symptom score; VAS, visual analogue scale.

As presented in Figure 1, the serum MIF levels were 20.7 ± 9.2 ng/ml in the AR group, which were markedly higher than those in the HC group (12.8 ± 4.7 ng/ml, *p* < 0.001), and MIF levels were significantly greater in the MSAR group (21.9 ± 9.8 ng/ml) than those in the MAR group (17.3 ± 6.5 ng/ml) and the HC group (12.8 ± 4.7 ng/ml, all *p* < 0.05). Spearman’s correlation analysis results showed that the serum levels of MIF in AR patients are positively correlated with TNSS (r = 0.599, *p* < 0.001), VAS (r = 0.505, *p* < 0.001), serum HDM–specific IgE (r = 0.7519, *p* < 0.001), total IgE (r = 0.221, *p* = 0.005), blood eosinophil count (r = 0.5639, *p* < 0.001), and percentage (r = 0.7589, *p* < 0.001; Figure 2).

### Predicting Clinical Response of Sublingual Immunotherapy From Serum Migration Inhibitory Factor

**FIGURE 3:**
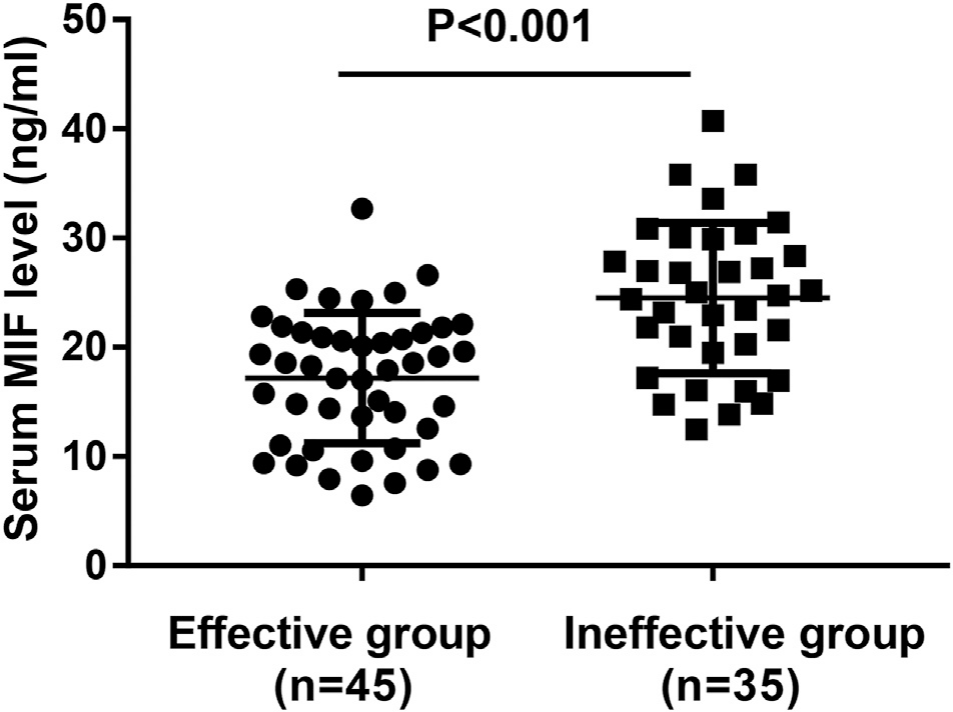
Comparison of serum MIF concentrations between the good-response group and the poor-response group. MIF, macrophage migration inhibitory factor.

**FIGURE 4:**
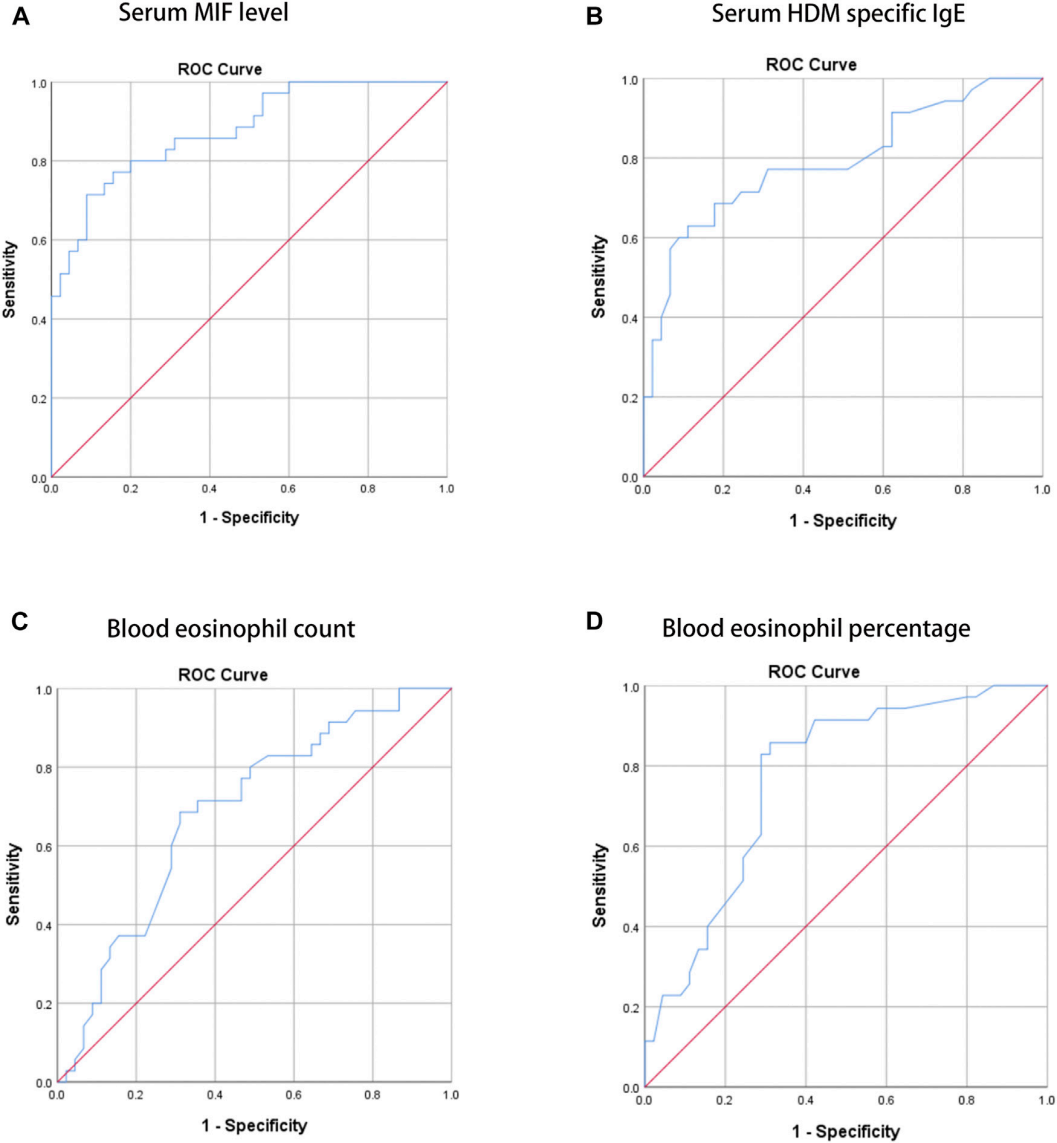
ROC analysis of serum MIF level **(A)**, serum HDM–specific IgE **(B)**, blood eosinophil count **(C)**, and percentage **(D)** in predicting the clinical response of SLIT in HDM-induced AR. ROC, receiver operating characteristic; MIF, macrophage migration inhibitory factor; HDM, house dust mite; AR, allergic rhinitis; SLIT, sublingual immunotherapy.

**FIGURE 5:**
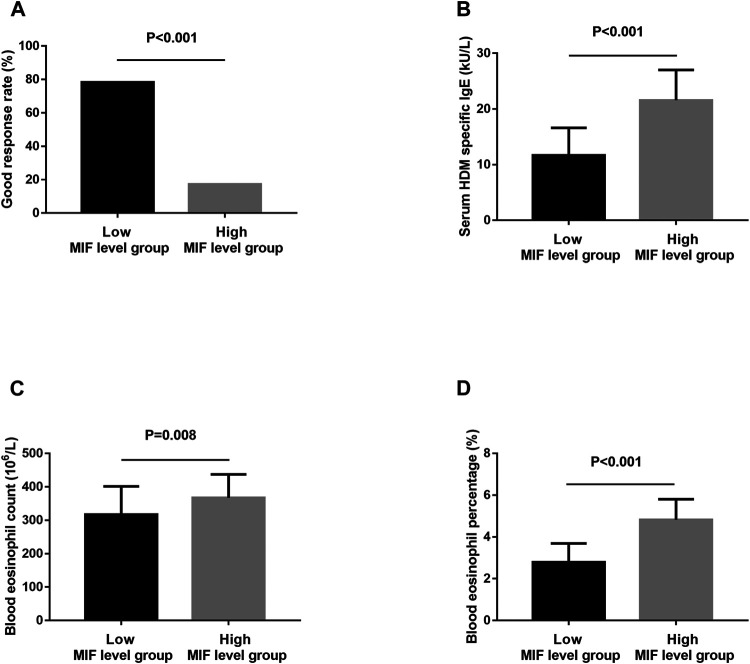
The good-response rate **(A)**, serum HDM–specific IgE **(B)**, blood eosinophil count **(C)**, and percentage **(D)** between the low MIF level group and the high MIF level group. HDM, house dust mite; MIF, macrophage migration inhibitory factor.

In the present study, 106 MSAR patients received SLIT, and 26 patients dropped out because of various reasons (Table 2), and the serum MIF levels in these 29 patients were 21.4 ± 7.9 ng/ml. During the treatment, 13 patients reported minor adverse events (Table 3) and no patient complained of major systemic adverse reactions. Finally, 80 MSAR patients successfully completed the whole course of SLIT and provided valid follow-up data: 45 patients were categorized into the good-response group and other 35 cases were grouped into the poor-response group. As Table 4 shows, the serum HDM–specific IgE, blood eosinophil count, and percentage in the good response group were significantly lower than those in the poor-response group (all *p* < 0.05), but no statistical difference was found in age, sex, disease duration, BMI, smoking, serum total IgE, TNSS, and VAS. The concentrations of serum MIF were markedly lower in the good-response group (17.9 ± 7.1 ng/ml) than those in the poor-response group (23.5 ± 8.8 ng/ml, *p* < 0.001; Figure 3). The ROC analysis results in Figure 4 showed serum MIF exhibited better accuracy and utility to predict clinical response of SLIT in AR patients than serum HDM–specific IgE, blood eosinophil count, and percentage. The detailed parameters of ROC analysis are displayed in Table 5. Based on the optimal cutoff values of the serum MIF level obtained by ROC analysis, patients were categorized into low MIF level group (serum MIF level <22.5 ng/ml, n = 51) and high MIF level group (serum MIF level >22.5 ng/ml, n = 29) as similarly described in previous publications (Liang et al., 2020; Wu et al., 2021). In low MIF level group, the good-response rate was 78.4%, which was higher than that in high MIF level group (17.2%, *p* < 0.001). In addition, the serum HDM–specific IgE, blood eosinophil count, and percentage were significantly lower in low MIF level group than those in the high MIF level group (all *p* < 0.05; Figure 5)

**TABLE 2:** Different reasons for the dropout during the SLIT.

Reason	N (%)
Poor clinical response	9 (8.5)
Loss to follow-up	9 (8.5)
Adverse events	3 (2.8)
Noncompliance	3 (2.8)
Pregnant	2 (1.9)
Total	26 (24.5)

SLIT, sublingual immunotherapy.

**TABLE 3:** Different minor adverse events during the SLIT.

Adverse event	N (%)
Pruritus and swelling of mouth, tongue, or eye	6 (5.7)
Aggravating rhinitis	3 (2.8)
Throat irritation	2 (1.9)
Gastrointestinal symptom	1 (0.9)
Nose bleeding	1 (0.9)
Total	13 (12.2)

SLIT, sublingual immunotherapy.

**TABLE 4:** Demographic and clinical variables between two groups.

Variable	Good-response group (n = 45)	Poor-response group (n = 35)	*p*-value
Age (year)	31.4 ± 8.1	31.3 ± 8.1	0.921
Sex, male (%)	22 (48.9)	16 (45.7)	0.824
Disease duration (year)	4.4 ± 1.7	4.5 ± 1.9	0.787
BMI (kg/m^2^)	22.7 ± 1.7	23.1 ± 1.9	0.325
Smoking (%)	9 (20.0)	10 (28.6)	0.433
Serum total IgE (IU/ml)	235.2 (102.4, 417.5)	268.1 (124.6, 501.2)	0.086
Serum HDM–specific IgE (kU/L)	10.3 (4.3, 17.5)	21.2 (9.8, 32.3)	<0.001
Blood eosinophil count (10^6^/L)	313.2 ± 84.2	364.0 ± 71.4	0.006
Blood eosinophil percentage (%)	3.0 ± 1.2	4.2 ± 1.2	<0.001
TNSS	9 (7, 11)	9 (7, 11)	0.849
VAS	7 (5, 9)	7 (5, 9)	0.916

BMI, body mass index; HDM, house dust mite; TNSS, total nasal symptom score; VAS, visual analogue scale.

**TABLE 5:** ROC analysis results of different variables for predicting clinical efficacy of SLIT.

Variable	AUC	SE	*p*-value	95%CI	Cutoff value	Sensitivity	Specificity
Serum MIF level (ng/ml)	0.877	0.038	<0.001	0.803–0.952	22.5	0.714	0.911
Serum HDM–specific IgE (kU/L)	0.791	0.053	<0.001	0.688–0.894	18.8	0.600	0.889
Blood eosinophil count (10^6^/L)	0.687	0.060	0.004	0.570–0.804	364.9	0.686	0.689
Blood eosinophil percentage (%)	0.771	0.053	<0.001	0.667–0.875	3.2	0.857	0.712

ROC, receiver operating characteristics; MIF, migration inhibitory factor; HDM, house dust mite; AUC, area under the curve; SE, standard error; CI, confidence interval.

### Multivariate Analysis of Variables Associated With Clinical Response of Sublingual Immunotherapy

Table 4 revealed the results of the possible factors associated with clinical response of SLIT in the univariate analysis, and variables with a *p* value <0.05 in the univariate analysis and serum MIF were further included in the multivariate logistic regression analysis. Unadjusted and adjusted multivariate analysis models both demonstrated that serum MIF was an independent factor associated with the clinical efficacy of SLIT (*p* < 0.001) (Table 6).

**TABLE 6:** Unadjusted and adjusted logistic regression models for predicting clinical efficacy of SLIT.

Variable	Unadjusted		Adjusted	
	OR (95% CI)	P	OR (95% CI)	P
Serum MIF level (ng/ml)	2.216 (1.489–3.197)	<0.001	2.834 (1.672–4.156)	<0.001
Serum HDM–specific IgE (kU/L)	1.893 (1.254–2.544)	0.002	2.030 (1.417–2.705)	0.030
Blood eosinophil count (10^6^/L)	1.219 (0.936–1.793)	0.389	1.467 (0.903–1.864)	0.401
Blood eosinophil percentage (%)	1.647 (0.924–2.098)	0.134	1.905 (0.917–2.699)	0.255

SLIT, sublingual immunotherapy; MIF, migration inhibitory factor; HDM, house dust mite; OR, odds rate; CI, confidence interval.

Adjusted for age, sex, disease duration, BMI, smoking, serum total IgE, TNSS, and VAS.

## Discussion

To the best of our knowledge, the present study was the first one to demonstrate that serum MIF levels were elevated in HDM-induced AR patients, and the increased MIF concentrations correlated with TNSS and VAS. On the other hand, the MIF levels were significantly lower in the serum of MSAR patients with good clinical response of HDM SLIT than those with poor clinical response. ROC and multivariate analyses showed that serum MIF was an ideal predictor for clinical response of SLIT in HDM-induced AR patients. These findings suggested that serum MIF appeared to be an important biological indicator for reflecting disease severity and an independent predictor to detect those who might best benefit from SLIT.

MIF is a unique pleiotropic protein which is mainly expressed on monocytes, macrophages, and lymphocytes, and serves as an important pro-inflammatory mediator and immune regulator that participates in innate and adaptive immune responses (Noe and Mitchell, 2020; Liu et al., 2021). Prior publications reported a pivotal role of MIF in the pathogenesis of type 2–mediated inflammation, especially in allergic and autoimmune diseases (Yoshihisa et al., 2011; de Souza et al., 2015; Bozza et al., 2020). A recent study observed that MIF amounts were increased in the allergen-induced skin tissue of atopic dermatitis murine models, and the elevated MIF levels promoted the secretion of IL-4 and IL-5, and eosinophil recruitment in the skin (Yoshihisa et al., 2011). Gamez-Nava and colleagues found that levels of MIF were higher in the serum of lupus nephritis patients than levels of MIF in that of HCs and positively correlated with the severity of proteinuria and renal dysfunction (Gamez-Nava et al., 2020). Although MIF is known as an important pro-inflammatory cytokine, the role of MIF in AR remains elusive. In the present study, we found that the concentrations of serum MIF increased in HDM-induced AR patients in comparison with those in HCs, especially in MSAR patients, and the elevated MIF concentrations correlated with TNSS, VAS, serum HDM–specific IgE, serum total IgE, blood eosinophil count, and percentage. Our current evidences are consistent with previous findings and indicate that MIF is essential in the pathophysiology of HDM-induced AR. Accordingly, recent publications highlighted that MIF was crucial in the effector phase of type-2 immune responses and positively regulated the polarization of macrophages and promoted the CD4^+^ T-cell differentiation to Th2 cells, improving the productions of Th2-type cytokines, then activated the B cells and secretion of IgE, and enhanced mast cell degranulation and histamine release, resulting in exacerbation of nasal symptoms (Bui et al., 2017; Bozza et al., 2020; Lan et al., 2020). Together, these findings suggested that increased serum MIF might associate with the occurrence and development of AR and be able to serve as an objective biomarker in reflecting the disease severity.

Although HDM SLIT has been proven to be effective and safe for moderate–severe HDM-induced patients, its efficacy varies across patients, and a certain proportion of users responded poorly to this treatment (Li et al., 2019; Drazdauskaitė et al., 2020). Presently, clinicians evaluate the efficacy of SLIT primarily by observing the changes of the subjective symptom score, which makes the clinical assessment somewhat inaccurate (Liu et al., 2020b). Identification and validation of accurate and reliable predictor for the clinical response of SLIT are urgently needed to achieve personalized treatment. In this study, we observed that the serum MIF levels were significantly decreased in the patients with good response to SLIT compared with those in patients with poor response. ROC and multivariate analysis results showed that serum MIF was reliable for predicting clinical response of SLIT, and exhibited better accuracy and utility than serum HDM–specific IgE, blood eosinophil count, and percentage. It was reported that downregulation of the Th2 inflammation with a shift toward Th1 response and modulation of specific IgE-mediated immune response were the underlying mechanisms of SLIT (Drazdauskaitė et al., 2020; Lam et al., 2020; Kloek et al., 2021). Prior studies showed that immunotherapy and corticosteroid treatments could reduce the circulating MIF levels and attenuate inflammatory responses *via* regulating the Th1/Th2 inflammation balance in nasal polyps and rheumatoid arthritis (Ekinci, 2018; Bilsborrow et al., 2019). Thus, we supposed that serum MIF mainly operated the activation of macrophage and promoted the differentiation of Th2 cells and its secretion, and facilitated the production of IgE, mast cell degranulation, and histamine release. Elevated levels of MIF in serum of poor responders can to a greater extent enhance the process of macrophage-driven Th2 inflammation, which implied that serum MIF may be involved in the SLIT and affect its clinical efficacy, and serum MIF may be clinically meaningful as an objective biomarker for predicting the clinical responsiveness of SLIT in HDM-induced AR patients.

In conclusion, serum MIF appeared to be a useful biological indicator for evaluating disease severity and an independent predictor for clinical response of SLIT in HDM-induced AR patients. Future studies with a larger sample number are required to confirm and extend our present findings.

## Data Availability

The raw data supporting the conclusions of this article will be made available by the authors, without undue reservation.
