# Supplementary material for: Circulating MIF Associated With Disease Severity and Clinical Response of Sublingual Immunotherapy in House Dust Mite–Induced Allergic Rhinitis
Source: Front Pharmacol. 2021 Jul 8;12:681724. doi: 10.3389/fphar.2021.681724 (PMC8296466; doi:10.3389/fphar.2021.681724)
Supplement: Supplementary file 1 [file Table1.DOCX]

| Severity | Score | Definition |
| --- | --- | --- |
| None | 0 | No symptom |
| Mild | 1 | Present but not bothersome |
| Moderate | 2 | Bothersome but tolerated |
| Severe | 3 | Severe and hard to tolerate |

Table S1 Four individual symptom scores in TNSS

TNSS, total nasal symptom score
